# Is ureteroscopy and active stone treatment safe and effective in octogenarians? A review of current literature

**DOI:** 10.1080/20905998.2024.2320028

**Published:** 2024-02-19

**Authors:** Daksh Bhatnagar, Carlotta Nedbal, Bhaskar Kumar Somani

**Affiliations:** a Department of Medical, University Hospital Southampton NHS Foundation Trust, Southampton, UK; b Department of Urology, University Hospital Southampton NHS Foundation Trust, Southampton, UK

**Keywords:** Ureteroscopy, kidney calculi, elderly, old, outcomes, urolithiasis

## Abstract

**Background:**

With the aging of our patient population, and the increasing incidence of kidney stone disease in the elderly, active stone treatment is becoming more common. In this review of current literature, we aim to assess safety and efficacy of ureteroscopy (URS) as primary treatment for urolithiasis in the octogenarians.

**Materials and methods:**

A scoping review of literature according to the PRISMA guidelines was performed, using the relevant search terms. Original articles were screened and included. A narrative review of the studies is provided, with emphasis on outcomes of URS in the elderly.

**Results:**

10 studies were included in the analysis. URS performed in the elderly population showed a good safety and efficacy, with stone-free rates (SFR) comparable to the general population. URS specific complication rates seems to be comparable to the other age groups, with postoperative events mostly related to anaesthesia and pre-existing medical conditions. The overall complication rate was still low, with a slightly prolonged hospital stay. Predictors for SFR were age, severe comorbidities and stone burden.

**Conclusion:**

URS for stone treatment in the elderly population is safe and effective, with comparable surgical outcomes to that of the general population. As comorbidities play an important role in the fitness for surgery and overall survival, risks and benefit of active stone treatment should be carefully balanced in this group.

## Introduction

An increasing elderly population presents with it a set of challenges due to physiological and anatomical differences compared to a younger age group. This is especially the case for urological disorders and impacts the subsequent management. The rising prevalence of diabetes and obesity, in conjunction with insufficient fluid intake and poor dietary control, contributes to the significant worldwide burden caused by kidney stone disease (KSD) [1]. Therefore, studies on the epidemiology of KSD has seen a rise in the incidence rates in the elderly population [2]. In particular, the cohort of over 75-year-old had the largest increase by 51% of finished consultant episodes (FCEs), compared with any other age group for KSD between 2006/2007 and 2013/2014 [3]. With demographic ageing, this provides a complex patient cohort with little previous research about the most appropriate and efficacious treatment.

Three treatment modalities, encompassing shockwave lithotripsy (SWL), ureteroscopy lithotripsy (URSL) and percutaneous nephrolithotomy (PCNL) [4] have all been proved to be safe and efficient in the treatment of urolithiasis in almost every age group. For ureteric stones of any size and for stones in the kidney up to 2 cm, for any location in the ureter or kidney, URSL is the first-line treatment for urolithiasis, as recommended by the European Association of Urology (EAU) guidelines [5]. However, there are limited studies demonstrating the differences in the outcomes of the general population and patients older than 80 years [6].

Advancements in research and technology provides a platform where better treatment options with higher levels of safety and efficacy are available, and knowledge about the trends for KSD enables higher specificity in stone management to achieve better outcomes in every age group [7]. However, the literature behind the evidence-based practice for one of the fastest-growing demographics in terms of age has been limited until recently [8], with only few studies reporting on management of urolithiasis in the elderly.

Compared with other options, URS has significantly fewer contra-indications and lower complication rates [9]. The continuous evolution of technology, with new smaller instruments and improved camera systems, expands the range of patients who can benefit from URS [10]. If anaesthetic risks, preoperative and postoperative management contribute to the complexity of KSD treatment in the elderly [11], advancements in these areas produces safer outcomes for the elderly population.

In the context of this growing burden for healthcare, we aim to review the literature regarding URS in the elderly, drawing a picture of the efficacy and safety of this well-known treatment option in the fragile octogenarian cohort.

## Materials and method

Two independent authors (CN, DB) performed a literature search through PubMed, Cochrane database and Google Scholar, and conflicts were resolved by a senior author (BKS). The review followed the Preferred Reporting Items for Systematic Reviews and Meta-analysis (PRISMA) methodology [12]. MeSH terms and keywords employed were as follows: ‘ureteroscopy’, ‘ureterorenoscopy’, ‘URS’, ‘retrograde intrarenal surgery’, ‘RIRS’, ‘elderly’ and ‘octogenarians’. Boolean operators (AND, OR) that were used to refine the search. Articles were first screened by title and full-text; full-text analysis was performed for articles eligible for inclusion from inception of databases to June 2023. Papers in the non-English language were excluded during the screening process.

All English-language original studies reporting on URS in population aged ≥80 years were considered eligible for inclusion. A threshold of a cohort of 25 patients was established for eligibility. Non-English articles, studies examining non-urolithiasis conditions, reporting on treatment of urolithiasis with other modalities then URS or in a population younger than 80 years old were excluded during the screening process. Case reports, review articles, historical cohort studies where data on URS could not be separated, laboratory studies, and animal studies were also excluded.

**Figure 1.**
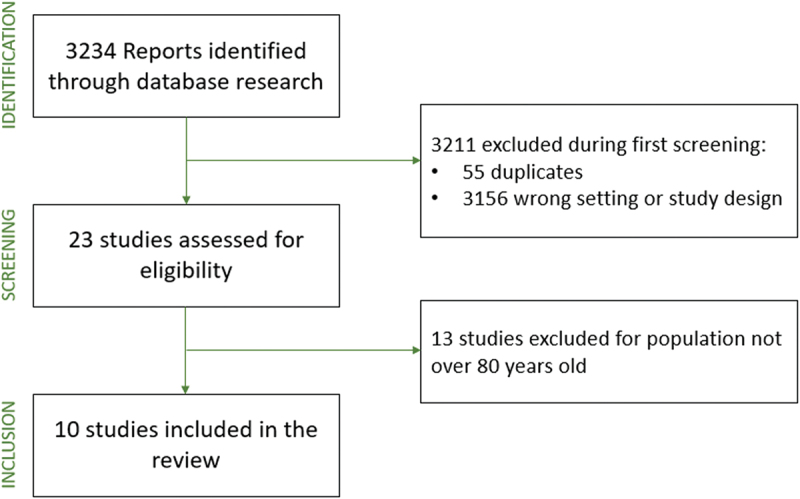
PRISMA flowchart of included studies.

Data were collected with Microsoft Excel. PRISMA chart shows the screening and inclusion process (Figure 1), retrieving 10 original studies [6,13–21].

Safety and efficacy were set as first outcomes, with data on SFR, complication rate and readmission rates recorded. Preoperative characteristics were collected, including age, gender, stone characteristics (size, location, number), Performance Status and/or American Society of Anesthesiologists (ASA) classification and comorbidities. Intraoperative features such as type of anaesthesia, surgical time, decision to leave a ureteric stent, and length of hospital stay were also recorded. We then included postoperative characteristics: SFR, stone composition, grade and rate of complications according to the Clavien Dindo Classification and rates of postoperative re-admissions.

## Results

### Preoperative patient characteristics

A total of 10 studies (1019 patients) were included, with the reviewed literature showing a degree of heterogenicity in the demographics of the reported patients. The sample size ranged from 27 to 338 across 10 studies, with a mean age of studies between 81 and 89 years, with the cut-off for the lower end being 80 years. Eight of these studies focused on URS independently [6,13–16,19–21] and the other two discussed URS in conjunction with SWL [17,18], under a common description of ‘active stone treatment’, rendering some of the data unsuitable for inclusion in certain categories, where data could not be retrieved separately.

The octogenarian cohort differed largely in male-to-female ratio amongst the studies, but overall, 47.15% males to 52.85% females were accounted for.

The stone size stayed relatively similar throughout the studies, with the mean ranging from 7.37 mm −13.4 mm. The stone density was reported in three of the studies [16,17,20] with a mean range of 570 HU to 940 HU. The stone location was in the ureter (*n* = 449), kidney (*n* = 413), with 38.5% patients having stones in multiple locations. Only 4 studies [13,15,17,18] commented on the Performance Status (PS), with 29.6% PS of 0 or 1, 32.5% PS of 2 or 3 and 37.9% PS of 4. Apart from one study [19], all other studies collected ASA scores. The ASA score of 1 was seen in 1.85%, 2 in 43.05% and 3 or more in 55.14%.

Common comorbidities were also accounted for within the data collection of most of the studies. There was a large variation between the studies but on average, 16% of the patients had diabetes, 40.4% had cardiovascular disease and 27% required medication for anticoagulation. A pre-operative stent or a nephrostomy was placed in 68% of the patients.

Table 1 summarises demographic and preoperative characteristics.

**Table 1.** Demographic and preoperative characteristics of the studies included.

			Gender			Stone location		Stone n°		ASA			Comorbidities				
Study	N° of patients	Age (years)	M (%)	F (%)	Stone size (mm)	Renal	Ureter	Single	Multiple	I	II	≥III	Diabetes (%)	Cardiovascular disease (%)	Anticoagulant drugs (%)	Preoperative positive urine culture	Preoperative drain
Aykac	29	87.31±2.33	58.6%	41.4%	16 (7–30)	20	9	N/A	N/A	0	1	28	N/A	N/A	N/A	N/A	N/A
Drerup	338	85.1±4.28	54.9%	45.1%	7.37 (1–35)	47	47	N/A	N/A	11	80	134	79.3%	89.7%	81.9%	15.2%	N/A
Emiliani	95	81 (80–94)	47.4%	52.6%	13.4±8.2	47	47	46	47	0	23	72	30.5%	58%	24%	N/A	79
Eredics	69	83.8 ±3.32	37.7%	62.3%	9.8±7.26	63%	37%	49%	51%	4.3%	20.1%	75.6%	24.4%	13.7%	43.9%	N/A	N/A
Juliebo-Jones	51	88 (85–97)	66.7%	33.3%	Ureteral 8 (3–27) Renal 13 (4–30)	20	53	Ureteral 87% Renal 57%	Ureteral 13% Renal 43%	0	12	52	16%	>58%	34%	N/A	27 (42%)
Koterazawa	166	85 (82–88)	35.5%	64.5%	10 (7.1–16)	131	114	36%	64%	5	125	36	24.1%	32%	N/A	38 (22.8%)	155
Sinha	94	85±3.9	70.6%	29.4%	13±8.2	48	54	92	25	N/A	N/A	N/A	N/A	N/A	N/A	N/A	45
Solomon	96	84	59.4%	40.6%	10	58	83	71%	29%	0	30	66	N/A	N/A	19%	41.6%	72%
Taguchi	27	87.8±5.5	14.8%	85.2%	11.4 ± 6.3	13	17	19	8	0	25	2	N/A	N/A	N/A	19	22
Tamiya	54	89 (85–101)	25.9%	74.1%	13.6±10 (total burden)	29	25	41	13	4	34	16	5.6%	32%	30%	77.8%	31

M: male; F: female; N/A: not applicable.

### Intraoperative features

The mean operating time for the procedures ranged from 45–102 minutes, but this is partly attributed to the fact that there was variation in defining the operating time. Most studies included the anaesthetic aspect of the procedure in the total time calculation.

Few studies reported the type of anaesthesia used, and in the ones that did, there was no concordance with the type used. One study used a spinal anaesthesia (SA) in 89% [13], with another having 37% SA and 33% general anaesthesia (GA) and 9% with sedation only procedure [16]. Two other studies [6,14] had all patients who underwent a GA. This alludes to the difference in approach within hospitals and countries.

The length of hospital stay was dependent on the complications intraoperatively and postoperatively. There was again a broad range of averages ranging from 0 to 10 days. Five of the studies had all of their patients with a post-operative stent after the procedure. Among the others, the lowest stent inserted rate was found in Juliebø -Jones’s study [16] of 66%, hence demonstrating a large majority of patient requiring stent insertion after URS.

Intraoperative and postoperative results are shown in Table 2.

**Table 2.** Intraoperative and postoperative results of the studies included.

		Anaesthesia							CR Clavien-Dindo					
Study	Operation time (min)	GA	SA	Sedation	Hospital stay (days)	Stent inserted	SFR (%)	Definition of SFR	I	II	III	>III	overall CR (%)	Re-admission rate
Aykac	45 (20–80)	10.3%	89.7%	0%	1 (1–5)	N/A	75.9%	Fragments <2mm at 30-days CT KUB.	N/A	12	0	0	41.4%	3.40%
Drerup	N/A	N/A	N/A	N/A	5.86	313 (93%)	80.0%	N/A	N/A	N/A	N/A	N/A	9.8%	N/A
Emiliani	102.96±45.51	100%	0%	0%	2.87±2.71	85	71.4%	Complete absence of fragments at 30 days on USS and Xray KUB.	1	8	0	0	9.5%	5.6%
Eredics	N/A	N/A	N/A	N/A	4.7	N/A	67.6%	N/A	N/A	10.1%	0	2	N/A	N/A
Juliebo-Jones	60 (15–120)	33%	37%	9%	2 (0–6)	42 (66%)	92% (A) 96% (B) 100% (C)	A: 0 fragment) B: =<2mm fragments). C: 2.1-4mm fragments. Non-contrast CT at 3 months	3	17	2	4	14% (intra-op) 13% (early) 28% (delayed)	27%
Koterazawa	62 (38.3–87.8)	N/A	N/A	N/A	7 (5–12)	100%	80.1%	Fragments <2mm at 30-days CT KUB.	1	29	1	3	20.5%	N/A
Sinha	47.06±25.7	N/A	N/A	N/A	0 ±7.1	79 (75.9%)	92.5%	Intra-operative finding OR fragments <2mm at 30-days imaging.	3	12	0	1	15.3%	N/A
Solomon	78±54	N/A	N/A	0%	N/A	100%	73.9%	Complete absence of fragments several weeks after surgery at CT or USS KUB.	15	6	1	4	44.5%	N/A
Taguchi	84±44.5	100%	0%	0%	10.1±6.7	100%	96.3%	Fragments <2mm at 30-days non-contrast CT.	1	0	0	0	3.7%	N/A
Tamiya	69.5±33.5	N/A	N/A	0%	N/A	100%	96.3%	Fragments ≤4 mm at 30-days CT. Xray or USS KUB.	N/A	N/A	2(≥3)	2(≥3)	18.1%	N/A

GA: general anaesthesia; SA: spinal anaesthesia; SFR: stone-free rate; CR: complication rate; N/A: not applicable; CT: computerised tomography; USS: ultrasound; KUB: kidneys-ureters-bladder.

### Postoperative

SFR showed largely positive outcomes with a range of averages from 71.4%-100%. The definition of SFR, disclosed in eight of the included studies, was harboured with different definitions. The threshold size of residual fragments varied from ≥4 mm, ≥2 mm to the absolute absence of any detectable fragment. Ultrasound (USS), computerised-tomography (CT) and X ray of the urinary tract (KUB) were differently performed in a time range between 30 days and 3 months from the URS procedure. The lowest SFR were seen with the strictest definition, with only one study reporting a SFR lower than 70%, as shown in Table 2.

The overall complication rate varied throughout the studies with the lower end being 3.7% [14] and the upper end being 44.5% [21]. The complications were categorised using the Clavien- Dindo (CD) system. Some of the studies grouped the categories differently from others and rendered the data less specific. The CD complications with grades 1, 2, 3 and 4 were seen in 5.8%, 13%, 0.99% and 2.48%, respectively. Individual complications were only addressed in 4 studies. Ureteric perforation and stent migration was managed by placement of a ureteric stent, fever and sepsis was managed with intravenous or oral antibiotics and acute urinary retention by placement of a urinary catheter.

The readmission rate was calculated for a few studies and ranged between 3.4% and 27%.

Only four of the studies included reported on the stone composition, with calcium oxalate accounting for more than a third, followed by struvite and uric acid.

The outcomes of each study are summarised in Table 3.

**Table 3.** Results and conclusion of the articles included in our review.

Study	Conclusion
Aykac, 2020	URS is effective and safe in geriatric patients, with unchanged surgical complications and SFR. A preoperative comprehensive evaluation in geriatric patients may help predicting and treating the complications.
Drerup, 2023	URS is safe and effective in octogenarians and nonagenarians. In elective settings, after having received urinary diversions, patients with reduced mobility and nonagenarians were less likely to undergo stone removal treatments.
Emiliani, 2021	Despite the higher rate of comorbidity in the elderly group, RIRS is safe with similar complication rate and outcomes at an expense of higher operative time and hospital stay.
Eredics, 2023	Age, frailty, performance-status and stone burden are predictors for active stone treatment. Octogenarians and nonagenarians, when fit for surgery, tend to live long enough to profit from active stone treatment.
Juliebo-Jones, 2023	The morbidity burden of URS in the extremely elderly is higher than for other population groups. Risk should be balanced carefully, considering conservative approach for high scores. Operation time should be kept to a minimum.
Koterazawa, 2023	URS for urolithiasis can be safely and effectively applied to octogenarians in selected cases.
Sinha, 2023	URSL is a safe procedure in the extremes of age groups with no difference in the overall outcomes between elderly and paediatrics.
Solomon, 2023	In elderly patients, URS for treatment of renal and ureteral stones is a relatively efficient and safe procedure, with low risk of major complications.
Taguchi, 2022	URSL is safe and effective for elderly people. Although oldest old people had multiple comorbidities with low performance status, URSL could be performed with acceptable complication rates.
Tamiya, 2023	URSL for older patients is as successfully as it is in young patients. Especially in older patients, preoperative stenting is an additional risk factor, which suggests that UTI may influence perioperative complications.

## Discussion

Our literature review finds that URS is safe and efficacious for the octogenarian cohort. The SFR and complication rates are comparable and not dissimilar from other younger age group, as particularly described by Sinha [19] and Tamiya [20]. Comparing URS data in the elderly population and in the young (<65 years old) or very young paediatric population (<10 years), both of the studies found that surgical complication rate and successful events were similar in the different age groups.

As demonstrated by the results of the studies included, postoperative complications in patients were often low grade and often noted to be a consequence of pre-existing comorbidities and anaesthesia, rather than the procedure itself. Among the studies, Juliebø-Jones and colleagues [16] reported the longer follow-up period, with overall complication rate at 3 months resulting higher than the rates reported at 30 days in the other studies. At the extremes of age, renal dysfunction and poor drug elimination were not uncommon [22]. As expected in this age group, general anaesthesia would produce greater side-effects such as delirium and post-operative confusion. This would suggest that clinicians need to work closely and consult with anaesthesiologists about the nuances of anaesthetising in this geriatric cohort.

As most of the postoperative complication seen in the studies included in our review might be reported to pre-existing medical conditions, an accurate pre-assessment is mandatory to plan an active intervention. When the overall general health of the elderly patients makes the procedure feasible, and the life expectancy is long enough, it has been shown that an active stone treatment can affect positively the quality of life, also reducing the risks of recurrent infections and sepsis related to an untreated urolithiasis [23–25].

URS has become more commonly used in the geriatric population and is proving to be more popular compared to the alternative options, namely SWL or PCNL, especially because a proportion of them will be on anticoagulants making these options less favourable compared to URS. In our review more than a quarter of patients were on anticoagulants.

Taguchi and colleagues [14] however preferred SWL for this age group due to its non-invasive nature. SWL is often contraindicated in patients who are on anticoagulation due to the risk of haematoma, and its success being dependant on clinical factors such as stone location and body habitus. A proximity of nearby vascular aneurysms could also have possibilities of rupturing during treatment [21]. Taguchi also commented on the limitations of patient’s habitus not being uniform [14]. Some patients had preoperative stenting which was mentioned to have possibly influenced the complication rate reported [26,27]. Due to their age and possibly some related dementia, anaesthesia related complications such as delirium could also be underreported. Similarly, the length of hospital stay might not solely be attributed to peri/postoperative complications but could be down to social circumstances, for example, housing or need for occupational or social care.

The definition of SFR varied amongst studies, with different modalities to assess it, and the timing of follow-up to assess it, and also how residual stones were defined and managed. What emerges from our review is that achieving an acceptable SFR without endangering the overall survival rate of octogenarians is a realistic outcome in URS. The main factors influencing outcomes were severe comorbidities, as they impact on the surgical and anaesthetic time, and features such as stone burden and composition. As analysed by Eredics [18], increasing number and dimension of stones in the renal collecting system were associated with a decreased SFR. Moreover, patients with large and/or multiple renal calculi also had a high risk of requiring additional interventions.

Ureteroscopy, due to the miniaturisation and improvement in technological instrumentation [28–30], is becoming safer regardless of patient’s age. Indeed, the presence of severe comorbidities and an acute presentation increases the risks of surgical intervention. In these scenario it might be better to have a less invasive approach such as stent or nephrostomy and plan for elective stone treatment once they are optimised. However, as discussed by Drerup and colleagues [17], stents and nephrostomies are often related to an increased risks of infections and encrustation. Any elective treatment should therefore be promptly offered in an adequate timeline after the initial temporising measure, as with delays the risks connected to URS and anaesthesia can increase exponentially.

The limitation of our review lies in the fact that some studies were retrospective, and the outcome measures reported were not standardised making it difficult to compare results. Also, some studies where data on URS could not be separated from other treatment modalities were excluded. For studies where we could, data were thoroughly analysed to try to overcome this bias analysing only URS outcomes. Two of these studies which gathered data from both SWL and URS [17,18] still favoured active treatment for good outcomes. There is also a variation in the selection criteria for patients available for studies. In most studies, there was insufficient clarity regarding instrument selection (semi-rigid and flexible ureteroscopy) based on stone location, and this is an aspect future studies should address. Further studies with evaluation of longer-term outcomes, recurrence rate and impact on overall survival could improve our understanding of the effect of URS in elderly people.

## Conclusion

URS for stone treatment in the elderly population is safe and effective, with comparable surgical complication rate and stone free rates as the general population. As comorbidities play an important role in fitness for surgery and overall survival, risks and benefit of active stone treatment should be carefully balanced. When feasible, an active stone treatment can increase quality of life, without decreasing overall survival.
